# Socio-Demographic and Clinical Differences in Subjects with Tuberculosis with and without Diabetes Mellitus in Brazil – A Multivariate Analysis

**DOI:** 10.1371/journal.pone.0062604

**Published:** 2013-04-24

**Authors:** Barbara Reis-Santos, Rodrigo Locatelli, Bernardo L. Horta, Eduardo Faerstein, Mauro N. Sanchez, Lee W. Riley, Ethel Leonor Maciel

**Affiliations:** 1 Lab-Epi UFES – Laboratory of Epidemiology, Universidade Federal do Espírito Santo, Vitória, Espírito Santo, Brazil; 2 Post-Graduate Programme in Epidemiology, Universidade Federal de Pelotas, Rio Grande do Sul, Brazil; 3 Post-Graduate Programme in Saúde Coletiva, Instituto de Medicina Social, Universidade do Estado do Rio de Janeiro, Rio de Janeiro, Brazil; 4 Departamento de Saúde Coletiva, Faculdade de Ciências da Saúde, Universidade de Brasília, Brasília, Brazil; 5 Division of Infectious Disease and Vaccinology, School of Public Health, University of California, Berkeley, California, United States of America; St. Petersburg Pasteur Institute, Russian Federation

## Abstract

**Background:**

Several studies have evaluated the relationship between diabetes mellitus (DM) and tuberculosis (TB), but the nature of this relationship is not fully understood. TB incidence may be influenced by immunosuppression from DM, but this association may be confounded by other clinical and socioeconomic factors. We aimed to assess socio-demographic and clinical differences in TB patients with and without DM.

**Methods:**

Using the Brazilian national surveillance system (SINAN), we compared 1,797 subjects with TB and DM with 29,275 subjects diagnosed with TB only in 2009. We performed multivariate analysis to identify factors associated with the presence of DM among TB patients.

**Results:**

Subjects with TB – DM were older; have initial positive sputum smear test (OR = 1.42, 95% CI 1.26–1.60), and were more likely to die from TB (OR = 1.44, 95% CI 1.03–2.01). They were less likely to have been institutionalized [in prison, shelter, orphanage, psychiatric hospital (OR = 0.74, 95% CI 0.60–0.93)]; developed extra pulmonary TB (OR = 0.62, 95% CI 0.51–0.75) and to return to TB treatment after abandonment (OR = 0.66, 95% CI 0.51–0.86).

**Conclusions:**

Prevalence of NCD continues to rise in developing countries, especially with the rise of elderly population, the prevention and treatment of infectious diseases will be urgent. DM and TB represent a critical intersection between communicable and non-communicable diseases in these countries and the effect of DM on TB incidence and outcomes provide numerous opportunities for collaboration and management of these complex diseases in the national public health programs.

## Introduction

In 1993, the World Health Organization (WHO) declared tuberculosis (TB) a global emergency. The treatment strategy with directly observed therapy was launched by WHO to improve detection and effective treatment and to reduce case-fatality by half [1]. However new challenges have arisen with emergence of multidrug resistant tuberculosis (MDR TB) and the epidemic of HIV infection and AIDS associated with TB. New strategies for TB control was thus launched, including the STOP TB Strategy and the Global Plan to Stop TB [2], [3]. However, other medical conditions have become increasingly recognized to hamper effective TB control [1], [4].

Population ageing, urbanization and associated lifestyle changes have propelled the rapid increase in rates of non-communicable diseases (NCD) and among these is diabetes mellitus (DM) [5]. DM, in particular, type 2 DM, is a global epidemic that emerged over last three decades as a consequence of the epidemic of obesity [5], [6]. DM depresses the immunologic response that facilitates the development of infectious diseases, including infection by *Mycobacterium tuberculosis*, the agent of TB [7]. The health burden associated with these disorders is high and may increase further as the incidence of DM increases [8], [9].

TB is the third cause of death among subjects with NCD, and among the NCD, DM one of the most important [1], [4]. The relationship between diabetes and TB has already been object of many investigations but the association between these two diseases is not fully understood [10], [11], [12]. A systematic review found consistent evidence that risk of TB is higher among diabetic subjects [13]. In addition, subjects with diabetes have poorer TB treatment outcome [14]. The results of these studies suggest that TB – DM and TB patients have some different characteristics that should be taken into account when dealing with the management of TB and DM.

A prospective study in Southern Mexico took into account properly confounding and found that TB – DM patients had a higher probability of treatment failure, recurrence and relapse [15]. Among Tanzanians’ patients, a study found that majority of TB patients with diabetes comorbidity were young and lean [16]. However, most of the previous analyses conducted so far have not consistently controlled for socio-demographic characteristics as well as patients’ underlying immunosuppressive and chronic comorbidities [13], [14]. New investigations should be cautious about such potential sources of confounding.

In this study, we aimed to assess the socio-demographic and clinical differences in subjects with tuberculosis with and without DM using the Brazilian national surveillance system.

## Patients and Methods

In 2009, Brazil had 84,691 TB cases reported to the Information System for Notification of Diseases (SINAN – Sistema de Informação de Agravos de Notificação) [17]. The SINAN was established in the 1990s by the Brazilian Ministry of Health to collect and disseminate public health information [18]. It represents the main information system from which data are extracted for epidemiological analyses, often complemented with data from other official national databases, such as the Mortality Information System (SIM), Hospital Information System (SIH; for inpatient care information), and the Surveillance System for MDR-TB [19]. All this systems are available in the website http://dtr2004.saude.gov.br/sinanweb/ [17]. Although for this particular study, data were obtained from Tuberculosis National Program at Minister of Health in order to have identified subjects to avoid replication and misclassification.

Several studies have evaluated SINAN’s ability to provide reliable information regarding TB and associated co-infections or co-morbidities [20], [21], [22], [23]. In general, these studies point to limitations in completeness of variables and underreporting of TB cases and TB deaths, but SINAN nevertheless has served as an important source of data for conducting population-based studies.

We compared subjects with TB and DM (TB – DM) with those who only had TB (TB). The SINAN also provided information on socio-demographic variables, presence of comorbidities, TB features and treatment, which we included as covariates.

The following socio-demographic covariates were evaluated: age (>20 years, 20–39 years, 40–59 years and ≥60 years), gender (male, female), skin color (white, black, browns and other (Asian and indigenous), school level (<4 years, 4 to 8 years, >8 years), area of residence (urban, rural or periurban) and whether the individual was institutionalized (i.e.: prison, shelter, orphanage, psychiatric hospital).

Regarding comorbidity, we assessed the presence of other non-communicable conditions (i.e.: mental disorders) and alcoholism. Reported unknown status regarding these conditions and absence of information were considered as missing values.

The covariates related to TB included the type of treatment that is classified as new TB cases (subjects with TB diagnosis at first time), relapse (subjects that completed a previous TB treatment and acquired TB a second time) and, return after abandonment (subjects that abandoned a previous TB treatment and returned to treatment). It also included TB presentation (pulmonary, extra pulmonary, pulmonary+extra pulmonary), localization of extra-pulmonary TB, tuberculin skin test (positive if higher than 10+ mm), existence of chest X-ray suspicious for TB, result of initial sputum smear test, result of initial culture examination, and result of initial histopathologic examination.

The inclusion of the subjects in directly observed therapy, number of contacts (refer to number of subjects whose were registered as contacts of subjects reported as TB case at SINAN) were also included as covariates. The occupational status of TB transmission defined as TB acquired at workplace (mainly determined by inadequate environments or conditions of work) and based on results of contact tracing procedures is another covariate. Concerning final treatment outcome, the subjects were classified as cured (those that completed the treatment and had at least two negative results of smear examination), abandoned (those that did not attend to regular appointments for more than 30 days), died (those that died during TB treatment), transferred out of treatment center (those that were transferred of health care center) and developed MDR TB (those that developed MDR TB during TB treatment).

Those subjects with missing information on DM status were excluded, as well as those reported to be HIV test positive, because HIV can also contribute to immunosuppression [1], [24], [25], which could cause confounding. Therefore, the present analyses excluded 8,144 subjects with missing information on DM status and 47,273 subjects who were either HIV positive or whose information on HIV status was missing. The final study sample consisted of 29,275 subjects of whom 1,797 had DM.

Pearson chi-square test was used to compare proportions and the Student’s t test was used to compare means. Covariates associated (p≤0.05) with the outcome of interest were included in a hierarchical logistic regression model.

In the hierarchical analysis, the following covariates were included: step 1 (age+school level+skin color); step 2 (variables retained from step 1+ Institutionalization+DOTS); step 3 (variables retained from step 2+ TB form+initial smear+smear 2nd month+X ray suspicious for TB+culture+histopathologic examination+contacts number); step 4 (variables retained from step 3+ treatment type); and step 5 (variables retained from step 4+ outcome).

In each step, those covariates associated with the outcome (p≤0.05) were retained in the model. These analyses were performed with Stata, version 12.0.

This study was approved by the ethical committee of Centro de Ciências da Saúde (Center of Health Sciences) of Universidade Federal do Espírito Santo – number 121/06.

## Results

In 2009, the prevalence of DM was 5.4% (95% CI, 5.2–5.5%) among 84,691 cases of TB reported by SINAN. We analyzed 29,275 subjects of whom 1,797 (6.1%; 95% CI, 5.9–6.4%) had TB – DM.

Gender was not associated with TB – DM. On the other hand, those subjects with TB – DM were older (mean age: 52±14 years) than those in the group with TB only (38±16 years) (p<0.001). The proportion of subjects with <4 years of schooling was the highest among those with DM – TB (p<0.001). Subjects identified as whites were more prevalent in the TB group (6.6%) compared to the TB – DM group (93.4%), (p = 0.025). TB – DM subjects had lesser institutionalization rates (Table 1).

**Table 1:** Distribution of socio-demographic characteristics of tuberculosis (TB) cases according to diabetes (DM) status in Brazil, 2009.

Characteristics (*)		TB – DM	TB	p**
		n (%)	n (%)	
Gender (29,273)	Female	609 (6.33)	9,008 (93.7)	0.334
	Male	1,188 (6.0)	18,468 (94.0)	
Age (29,010)	<20 years	20 (0.9)	2,132 (99.1)	<0.001
	20–39 years	258 (1.9)	13,402 (98.1)	
	40–59 years	980 (9.9)	8,900 (90.1)	
	≥60 years	524 (15.8)	2,794 (84.2)	
Skin color (25,621)	White	767 (6.6)	10,829 (93.4)	0.025
	Black	175 (5.4)	3,079 (94.6)	
	Browns	598 (5.9)	9,557 (94.1)	
	Other	34 (5.5)	582 (94.5)	
School level(16,318)	<4 years	352 (8.2)	3,960 (91.8)	<0.001
	4 to 8 years	398 (6.3)	5,896 (93.7)	
	>8 years	244 (4.8)	4,787 (95.2)	
	Not applicable	49 (7.2)	632 (92.8)	
Area of residence (17,791)	Urban	1,052 (6.6)	14,922 (93.4)	0.186
	Rural/periurban	105 (5.8)	1,712 (94.2)	
Institutionalization (28,122)	No	1,626 (6.4)	23,711 (93.6)	<0.001
	Yes	92 (3.3)	2,693 (96.7)	
Alcoholism (29,029)	No	1,458 (5.9)	23,028 (94.1)	0.751
	Yes	265 (5.8)	4,278 (94.2)	

(*)number of valid observations.

** Pearson chi-square test.

Table 2 describes the study subjects according to characteristics of TB presentation. Return for TB treatment after abandonment was greater among those who did not have DM (3.8% TB – DM vs 96.2% TB p<0.001). The proportion of subjects who had a positive tuberculin skin test was similar between the two groups.

**Table 2:** Distribution of presentation and treatment characteristics of tuberculosis (TB) cases according to diabetes (DM) status in Brazil, 2009.

Characteristics (*)		TB – DM	TB	p**
		n (%)	n (%)	
Treatment type (27,543)	New case	1,490 (6.2)	22,655 (93.8)	<0.001
	Relapse	119 (6.8)	1,638 (93.2)	
	Return after abandonment	62 (3.8)	1,579 (96.2)	
Tuberculin skin test (4,993)	Negative	111 (5.7)	1,842 (94.3)	0.526
	Positive	186 (6.1)	2,854 (93.9)	
X ray suspicious for TB (25,243)	No	74 (4.0)	1,788 (96.0)	<0.001
	Yes	1,524 (6.5)	21,857 (93.5)	
TB form (29,275)	Pulmonary	1,601 (6.6)	22,616 (94.4)	<0.001
	Extra pulmonary	156 (3.7)	4,071 (96.3)	
	Pulmonary+Extra pulmonary	40 (4.8)	790 (95.2)	
Initial smear (24,396)	Negative	417 (5.2)	7,553 (94.8)	<0.001
	Positive	1,144 (7.0)	15,282 (93.0)	
Culture (6,510)	Negative	106 (4.5)	2,265 (95.5)	0.022
	Positive	240 (5.8)	3,899 (94.2)	
Histopathologic examination(3,399)	AARB positive	75 (6.7)	1,043 (93.3)	0.013
	Suggestive	92 (4.4)	1,978 (95.6)	
	Not suggestive	15 (7.1)	196 (92.9)	
Smear 2^nd^ month (9,758)	Negative	499 (6.1)	7,687 (93.9)	<0.001
	Positive	139 (8.8)	1,433 (91.2)	
DOTS (23,737)	No	835 (6.5)	11,914 (93.5)	0.005
	Yes	800 (5.7)	13,188 (94.3)	
Occupational (15,426)	No	958 (6.4)	14,004 (93.6)	0.148
	Yes	22 (4.7)	442 (95.3)	
Outcome (17,750)	Cure	441 (3.3)	12,826 (96.7)	<0.001
	Abandonment	61 (3.99)	1,513 (96.1)	
	Death from TB	44 (7.6)	535 (92.4)	
	Another cause of death	29 (5.9)	460 (94.1)	
	Transfer of treatment center	124 (7.0)	1,653 (93.0)	
	MRTB	4 (6.2)	60 (93.8)	

(*)number of valid observations;

** Pearson chi-square test.

Prevalence of extra-pulmonary TB was 3.7% among TB subjects. The pleural TB was reported for 38% of TB – DM and 47% of TB subjects (p = 0.009).

Smear test was less likely to be performed among TB subjects but we believe that it did not change the results. The proportion of smear positive TB was 7% for the subjects with TB – DM and 93% for those with TB (p<0.001). Follow-up smear examination at the second month of treatment showed positive results in 8.8% of TB – DM and 91.2% of TB patients (p<0.001).

Subjects with TB – DM were less covered under the DOTS program (5.7% TB – DM vs 94.3% TB, p = 0.005).

There were no differences in occupational TB rates and the mean number of contacts was 3±3 persons for those with TB – DM and 3±5 persons for those with TB (p = 0.004).

The hierarchical multivariate model (Table 3) showed that those subjects in the age group 40–59 years and those ≥60 years were more likely to develop TB – DM. The odds of having TB – DM was also higher among those with positivity of initial sputum smear (OR = 1.42, 95% CI 1.26–1.60); and death from TB as treatment outcome (OR = 1.44, 95% CI 1.03–2.01).

**Table 3:** Hierarchical* multivariate analysis of the association of diabetes (DM) status and tuberculosis (TB) subjects characteristics in Brazil, 2009.

		Characteristics	OR**	95% CI
Step 1	Age (years)	<20 years	1.00	–
		20–39 years	2.05	1.30–3.24
		40–59 years	11.74	7.52–18.32
		≥60 years	19.99	12.75–31.36
Step 2	Institutionalization	No	1.00	–
		Yes	0.74	0.60–0.93
Step 3	TB form	Pulmonary	1.00	–
		Extra pulmonary	0.61	0.50–0.75
		Pulmonary+Extrapulmonary	0.76	0.55–1.06
	Initial smear	Negative	1.00	–
		Positive	1.42	1.26–1.60
Step 4	Treatment type	New case	1.00	–
		Relapse	0.88	0.72–1.08
		Return afterabandonment	0.66	0.51–0.86
Step 5	Outcome	Cure	1.00	–
		Abandonment	0.93	0.71–1.24
		Death from TB	1.44	1.03–2.01
		Another causeof death	1.09	0.73–1.62
		Transfer oftreatment center	1.22	0.99–1.52
		MRTB	1.25	0.44–3.56

* The hierarchical model included:

Step 1: age+school level+skin color.

Step 2: step 1+ Institutionalization+DOTS.

Step 3: step 2+ TB form+initial smear+smear 2^nd^ month+X ray suspicious for TB+culture+histopathologic examination+contacts number.

Step 4: step 3+ treatment type.

Step 5: step 4+ outcome.

** Adjusted odds ratio.

On the other hand, the TB – DM subjects were less likely to be institutionalized (OR = 0.74, 95% CI 0.60–0.93); to receive treatment after abandonment (OR = 0.66, 95% CI 0.51–0.86); and to develop extra-pulmonary TB (OR = 0.61, 95% CI 0.50–0.75).

## Discussion

According to the World Health Organization about 10% of TB cases globally are linked to diabetes [26]. In our study, the prevalence of DM among TB patients based on those reported in 2009 in Brazil was 5.4% (95% CI, 5.2–5.5%). In countries with similar burden of TB, such as Mexico [27], India [28], and Tanzania [29], the prevalence was 2.7%, 18.4%, and 6.7% respectively. On the other hand, in low-burden countries, such as Canada [30] and Finland [31], the prevalence was 0.14% and 14.6% respectively.

Our results found that subjects with TB – DM tended to be older; have more comorbidities, such as hypertension, respiratory diseases, mental disorders, cancer; developed pulmonary TB; have initial positive sputum smear test and have higher mortality. They were also less likely have been in prison, shelter, orphanage, and psychiatric hospital, and return to TB treatment after abandonment.

Subjects with TB – DM have been reported to have more severe TB and worse prognosis [14], [24], [27], [31], [32], [33]. This tendency to an unfavorable outcome was identified in our study, where mortality from TB was significantly higher in those with TB – DM. Immunological disturbance such as reduction in alveolar macrophages activation and in interleukin 10 production has been described in TB – DM subjects [34], [35]. On the other hand, a recent prospective cohort study found high rates of mortality from causes other than TB among patients with DM and this study also found high independent risks of recurrence and relapse in TB – DM subjects [15].

Although the biological basis for the association between both diseases is not fully understood, studies have suggested that DM depresses the immune response, which can facilitates infection with *Mycobacterium tuberculosis* and progression to disease after infection [1], [13], [24], [36], [37].

In contrast, the association between HIV infection and TB is well known. HIV infection leads to impaired phagocytosis and T cell immunity and is the strongest risk factor for TB [1], [24]. Therefore to avoid confounding in this study, subjects with positive HIV infection status were excluded.

However, some limitations should be mentioned. First, we may have underestimated the prevalence of DM because 8,144 subjects were without information on DM status. Second, missing other data was not negligible. Nevertheless, our large sample size still allowed us to maintain a high statistical power.

Another limitation was that information on smoking status and drug abuse, important risk factors for both conditions, is not regularly gathered by SINAN. Only 853 patients were reported as smokers. Among those, 43 had DM and the prevalence of smokers among TB – DM patients was 5% (95% CI, 4–7); because of these small numbers we did not include this variable in the analysis.

Similarly, the SINAN database does not include culture and drug susceptibility test results at second month and the reasons for not performing second month smear examination. In Brazil, *M. tuberculosis* culture is not routinely performed for all patients; culture and drug susceptibility tests are only recommended for special cases such as retreatment after failure, relapse, patients with suspected primary resistance and case contacts of a resistant TB case [38]. In our data only 31% of the patients samples were tested by culture at diagnosis.

The strengths of our study are its large sample size, the utilization of data based on an information system whose quality was confirmed in previous studies [19], [22], and covariates stratified by socio-demographic and clinical characteristics.

In our study, the likelihood of TB – DM was higher among older subjects. Despite the fact that most previous studies did not examine the role of age on the relationship between TB and DM [13], there are indications that subjects with TB – DM are 10–20 years older than those with TB [24], [39], [40]. The limitations of these data are that we did not examine subjects with DM only; those with type 2 DM without TB may be older than those with TB only.

The association of TB – DM with skin color and school level vanished in the adjusted model, similarly to what has been reported in a systematic review and a previous investigation [13], [41]. On the other hand the subjects’ institutionalization was inversely associated with TB – DM.

Chronic non-communicable diseases such as hypertension, other cardiovascular diseases, respiratory diseases, renal diseases, mental disorders, cancer often coexist [42]. In a recent report, it was also recognized that TB is only one of several possible ailments that DM patients face [43].

We found that returning for TB treatment after abandonment was less likely occur among subjects with TB – DM (OR = 0.66, 95% CI 0.51–0.86). This is supported by a previous cohort study in Rio de Janeiro, which showed a relative risk of 0.39 for treatment abandonment in subjects with both TB and DM [44]. This observation may reflect the fact that patient with DM have a higher frequency of medical care encounters for their DM, which may lead to resumption of TB treatment.

Our study shows that TB – DM patients were no more likely than those with TB to develop extrapulmonary TB. DM does not appear to influence extrapulmonary TB. On presentation, subjects with TB – DM reported more symptoms but had no evidence of severe disease. Smear examination at second month of treatment is an important marker of response to TB treatment. However, these data are contradictory in the literature: some studies suggest that diabetic subjects have lower conversion [45], [46], but other studies [47], [48] found no large difference, suggesting that those with TB – DM may have the same response to treatment.

The rate of TB treatment failure in Brazil is high, approximately 20%, regardless of patient’s DM status [49], [50], [51], [52]. It is important to note that only 64 subjects developed multidrug-resistant TB in our sample, and it was not associated with DM status.

In general DM is diagnosed before TB develops and this should be taken into account in the management of TB [53]. The connections between general primary care and TB control programs should be stronger. The risk factors we identified to be associated with DM – TB should be taken into consideration when dealing with TB patients. Opportunities for preventing poorer outcomes in this group should be addressed.

We suggest cost-effectiveness studies to assess if all confirmed TB patients should be systematically screened for DM as they are screened for HIV infection and if all DM patients should be screened for TB, as already suggested by Sullivan & Amor (2012) [54]. This strategy should have an impact on reducing incidence of TB and may contribute to the WHO goals for TB control [1], [4].

Finally, assuming that prevalence of NCD continues to rise in developing countries, especially with the rise of elderly population, the prevention and treatment of infectious diseases will be urgent. DM and TB represent a critical intersection between communicable and non-communicable diseases in these countries and the effect of DM on TB incidence and outcomes provide numerous opportunities for collaboration and management of these complex diseases in the national public health programs.
